# Percutaneous transhepatic recanalization of occluded prosthetic graft after pancreatoduodenectomy with venous reconstruction for pancreatic cancer

**DOI:** 10.3389/fonc.2025.1575481

**Published:** 2025-07-17

**Authors:** Nils Degrauwe, Didier Roulin, Vincent Dunet, Nermin Halkic, Nicolas Demartines, Antonia Digklia, Nicolas Villard, Alban Denys, Georgia Tsoumakidou, Rafael Duran

**Affiliations:** ^1^ Department of Radiology and Interventional Radiology, Lausanne University Hospital and University of Lausanne, Lausanne, Switzerland; ^2^ Department of Oncology, Lausanne University Hospital and University of Lausanne, Lausanne, Switzerland; ^3^ Department of Visceral Surgery, Lausanne University Hospital and University of Lausanne, Lausanne, Switzerland

**Keywords:** pancreatic cancer, pancreatoduodenectomy, venous graft thrombosis, venous resection, recanalization, survival

## Abstract

**Introduction:**

To investigate the feasibility, safety, and efficacy of percutaneous transhepatic endovascular recanalization and stenting after venous graft thrombosis in pancreatic cancer patients who underwent pancreatoduodenectomy (PD) with venous reconstruction and assess risk factors of occlusion.

**Methods:**

This retrospective study was approved by the institutional review board. The clinical characteristics of 227 patients who underwent PD were compared among patients who underwent PD with/without porto-mesenteric venous resection (PMVR) ± prosthetic graft interposition.

**Results:**

Out of 227 patients, 18 (8%) underwent PD with PMVR and prosthetic graft interposition. Seven out of 18 patients had prosthetic graft occlusion. Occlusion was symptomatic in most cases (86%) and associated with tumor recurrence in 43%. On univariable logistic regression analysis, small postoperative graft diameter (OR: 0.141; 95% CI 0.021–0.970) and caudal anastomosis diameter measured on CT (OR: 0.226; 95% CI 0.059–0.859) were clear predictors of graft occlusion (*p* = 0.047 and *p* = 0.029, respectively). Interventional recanalization was performed in five patients. Technical success was 100%, with no complications.

**Discussion:**

Percutaneous transhepatic prosthetic graft recanalization and stenting is feasible and may be considered a safe and effective technique with immediate restoration of porto-mesenteric blood flow and symptom relief. Small grafts and venous anastomosis diameters are particularly at risk of thrombosis.

## 1 Introduction

Surgery remains the only cure for pancreatic cancer. However, only 15%–20% of patients are surgical candidates since the disease is often diagnosed at an advanced stage with significant vascular invasion (1). Pancreatoduodenectomy (PD) with porto-mesenteric venous resection (PMVR) has been increasingly performed to expand the pool of patients eligible for surgery (2–6). Most patients undergo direct end-to-end anastomosis (46%) followed by venorrhaphy/patch (34%), while approximately 19% require interposition grafts (prosthetic or autologous) (3). Venous reconstruction thrombosis after PD was found to occur in 28.3% of patients and may develop early (<90 days from surgery; 7.5%) or late (>90 days from surgery; 20.8%), with local tumor recurrence being implicated in most cases in the latter scenario (7). Graft interposition is associated with reduced overall patency compared to PMVR with end-to-end anastomosis (8). When present, porto-mesenteric thrombosis is symptomatic in most patients (7, 8). Post-operative thrombosis management is highly variable, and anticoagulation therapy may be administered (9). Few reports have described endovascular techniques for porto-mesenteric venous occlusion (10–14). Although experience in different clinical scenarios has been obtained, whether this can be extrapolated to oncologic patients with a venous graft remains elusive. Our study aims to investigate the feasibility, safety, and efficacy of percutaneous transhepatic endovascular recanalization and stenting after venous graft thrombosis in pancreatic cancer patient who underwent PD with venous reconstruction and assess risk factors of occlusion.

## 2 Material and methods

This retrospective, single-institution study was approved by the institutional review board (CER-VD 2020-00260). Patient consent was waived due to the retrospective nature of the study in cancer patients with poor prognosis.

### 2.1 Study design

**Figure 1:**
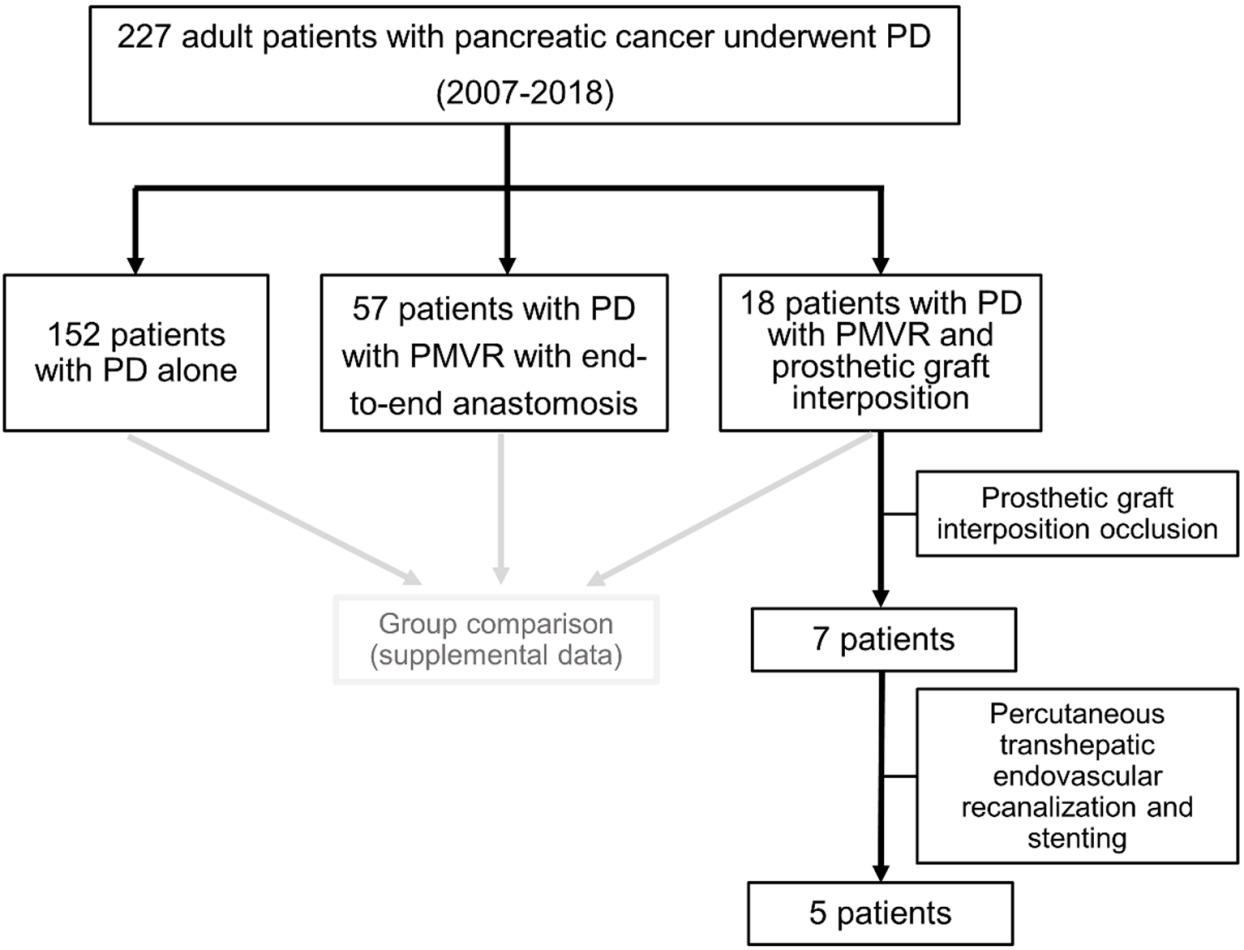
Flowchart of the study patients. PD, pancreatoduodenectomy; PMVR, porto-mesenteric venous resection.

A prospectively collected and maintained database of PD patients (who underwent surgery between 2007 and 2018) was used. Demographics, clinical, and pre-/intra-/postoperative data were obtained from medical records/prospective databases. Graft characteristics were obtained from follow-up CT. Patients were classified and compared among subgroups of patients who underwent PD alone, PD with PMVR with end-to-end anastomosis, and PD with PMVR and prosthetic graft interposition (**Figure 1**).

### 2.2 Surgical technique

A multidisciplinary tumor board discussed the cases of the patients, and patients gave their written informed consent to undergo the surgery. Patients were operated on by experienced surgeons who specialize in pancreatic surgery according to standard procedures. In addition, venous resection was performed if venous tumor infiltration was observed on preoperative imaging or suspected intraoperatively. The decision between end-to-end anastomosis and graft interposition was made by the surgeons. In cases where the length of the resected venous segment was short (commonly <6 cm), there was no significant tension on the anastomosis, and the residual vein ends were well mobilized, direct end-to-end anastomosis was performed. If needed, the distance between the venous anastomoses could be decreased by mobilizing the liver, detaching it from its diaphragmatic attachments. For the other cases, prosthetic graft interposition using polytetrafluoroethylene (PTFE) was performed (15–18). No autologous grafts were used. After surgery, prophylactic intravenous heparin was administered (at a dose of 10–15 units/kg/hour, targeting an anti-Xa level of approximately 0.2 IU/mL), followed by prophylactic subcutaneous low-molecular-weight heparin until discharge (typically 5,000 units bid). Graft patency was assessed on imaging during the postoperative stay via CT scan.

### 2.3 Percutaneous revascularization of occluded grafts

**Figure 2:**
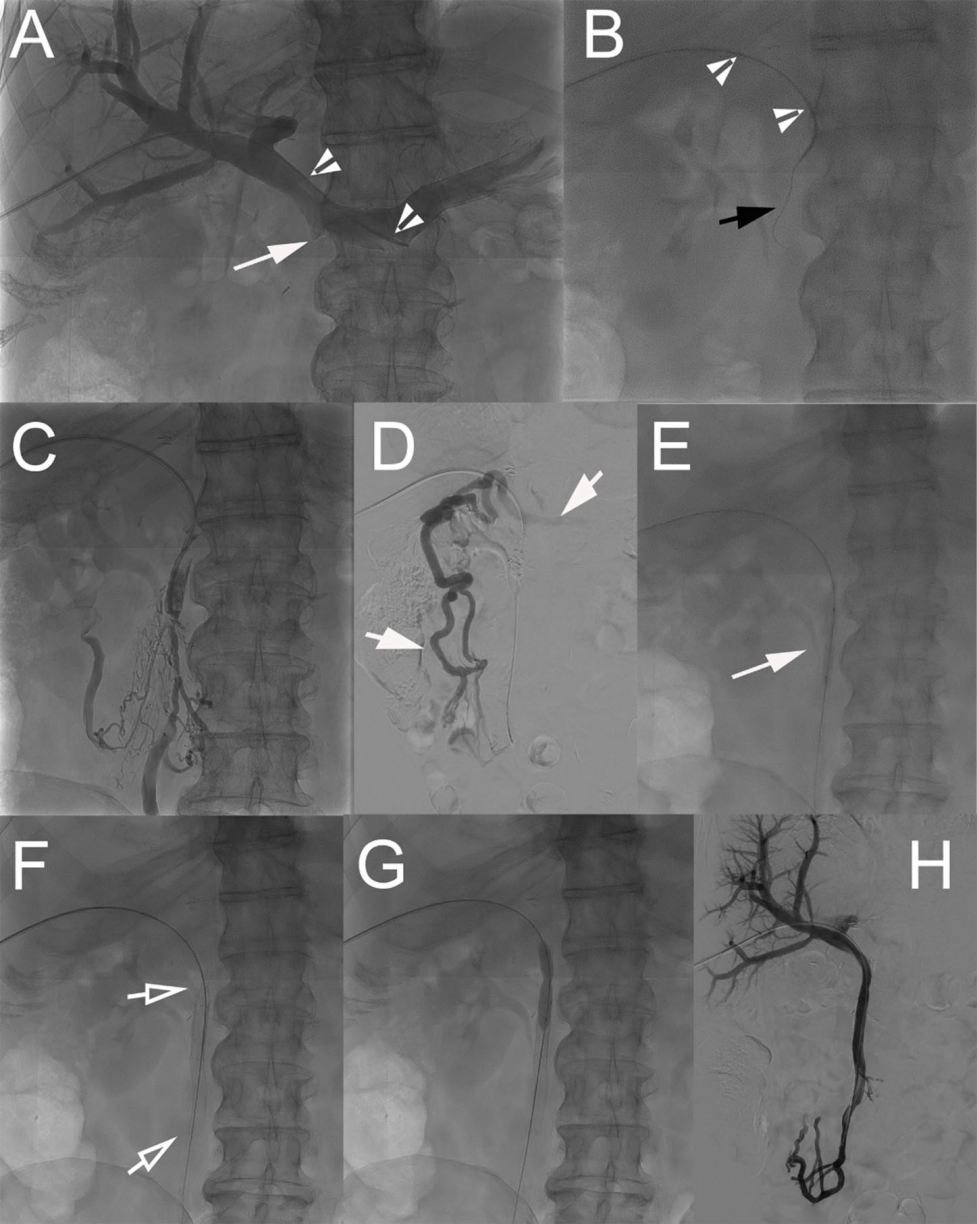
Technique of percutaneous transhepatic revascularization of occluded prosthetic graft. **(A)** Percutaneous transhepatic access to the portal system was performed through a puncture of a peripheral branch of segment V. A 5-Fr Berenstein catheter (arrowheads) was advanced in the splenic vein, and direct splenoportography was performed. The graft from the SMV was completely occluded; a small notch showed the location of the vein (arrow). **(B)** The occlusion was crossed with the catheter (arrowheads) in combination with a 0.035-in. stiff hydrophilic guidewire (black arrow). **(C)** After crossing the occlusion, gentle intrastent injection of contrast material was performed to confirm proper positioning. **(D)** Venography from an ileal branch of the SMV shows the absence of blood flow through the SMV and presence of dilated venous collaterals (short arrows). **(E)** The occluded graft and its anastomoses were dilated at 6 mm using a balloon catheter. **(F)** Two nitinol self-expanding stents [8 × 80 mm (Absolute Pro, Abbott) and 8 × 60 mm (SMART Control, Cordis)] were then placed (hollow arrows) and **(G)** balloon-dilated. **(H)** Completion venography showed complete recanalization of the SMV system with no signs of residual portal hypertension. SMV, superior mesenteric vein.

A multidisciplinary tumor board discussed the cases of the patients, and patients gave their written informed consent. Procedures were performed with the patients under general anesthesia by three interventional radiologists (with 5–20 years of experience). The peripheral right portal vein (PV) branch was punctured with ultrasound guidance using a 21 G Chiba needle followed by the placement of the Neff introducer set (Cook Medical, Bloomington, IN, USA). A 0.035-in. hydrophilic guidewire (Glidewire, Terumo, Japan) was then introduced, which allowed for the placement of an 8-Fr vascular sheath. A 5-Fr Berenstein catheter was advanced in the PV above the occluded graft. Portography and pressure measurements were performed (**Figure 2A**). Then, the occlusion site was crossed with the combination of the Berenstein catheter and hydrophilic guidewire. After crossing the occlusion, contrast material was injected to delineate the anatomy and determine the extent of thrombosis (**Figures 2B–D**). Pressure measurements were performed. The guidewire was then replaced with an Amplatz Super Stiff (Boston Scientific, Marlborough, MA, USA). The occluded segment was first dilated using a 6 mm/40 mm balloon catheter (Passeo-35, Biotronik, Berlin, Germany) (**Figure 2E**), and nitinol self-expanding stents (8–12 mm) (SMART Flex/Control, Cordis, Miami Lakes, FL, USA, and Absolute Pro, Abbott, Abbott Park, IL, USA) were placed and balloon-dilated (**Figures 2F, G**). Venography and pressure measurements were repeated (**Figure 2H**). The hepatic access point/track was embolized using microcoils (Tornado-5/3 mm, Cook) through the vascular sheath (ultrasound/fluoroscopic guidance). Postprocedural stenting patency was assessed via US and subsequently during follow-up via CT (every 3 months).

### 2.4 Statistical analysis

Data were summarized using descriptive statistics (mean and range for continuous variables and number and percentage for categorical variables). Three groups (PD and PMVR with prosthesis graft, PD and PMVR with end-to-end anastomosis, and PD without porto-mesenteric venous resection) were compared using the Kruskal–Wallis test for continuous variables and Fisher’s exact test for categorical variables. Logistic regression analysis with computation of odds ratio (OR) and 95% confidence intervals (95% CI) was additionally performed to evaluate potential predictors of surgical graft occlusion. Backward and forward stepwise multivariable logistic regression analyses were used to identify potential independent predictors of prosthetic graft occlusion. Only variables that demonstrated a *p*-value <0.15 on univariable logistic regression were included in this analysis. The *p*-value for removing from or adding to the model any variable was set at 0.10. Overall survival (OS) was defined as the date of surgery until death (or the last available follow-up). Survival curves were estimated using the Kaplan–Meier method and plotted for each group; differences were assessed using the log-rank test. A *p*-value <0.05 was considered statistically significant. Statistical analysis was performed using Stata 16 (StataCorp, College Station, TX, USA) and GraphPad Prism (v9, GraphPad Software, San Diego, CA, USA).

## 3 Results

### 3.1 Patient data

A total of 227 consecutive patients (119 men and 108 women; mean: 69 ± 11 years) were included. Of the patients, 152 (67%) underwent PD alone, 57 (25%) PD with PMVR with end-to-end anastomosis, and 18 (8%) PD with PMVR and prosthetic graft interposition. Seven of 18 patients had graft occlusion. Interventional recanalization was performed in five patients (**Figure 1**).

Patient characteristics are summarized in **Table 1**. A majority of patients from the PD with PMVR and prosthetic graft interposition group were female (56%). Eastern Cooperative Oncology Group (ECOG) performance status was 0, 1, and 2 in 11 (61%), 6 (33%), and 1 (6%) out of 18 patients. The mean tumor size was 3.9 cm (range: 2.4–6 cm), and the mean pre-operative CA19–9 was 764 kU/I. Five of 18 (28%) patients received neoadjuvant chemotherapy (28%), which was significantly more frequent when compared to the other PD subgroups (*p* = 0.002). Median whole cohort follow-up was 20.4 months (range: 0.13–107.5 months).

**Table 1:** Pancreatic cancer patient characteristics.

	Full cohort		PD + PMVR with prosthesis graft (N = 18)		PD + PMVR with end-to-end anastomosis (N = 57)		PD without PMVR (N = 152)		*p*-Value
	(N = 227)								
Age in years (mean and SD)	66	11	67	11	67	10	66	11	0.724
Sex									
Male	119	(52%)	8	(44%)	26	(46%)	85	(56%)	0.330
Female	108	(48%)	10	(56%)	31	54%	67	(44%)	
ECOG performance status									
0	85	(37%)	7	(39%)	22	(38.5%)	56	(37%)	0.800
1	83	(37%)	5	(28%)	22	(38.5%)	56	(37%)	
2	20	(9%)	2	(11%)	3	(5%)	15	(10%)	
3 and above	2	(1%)	0	(0%)	1	(2%)	1	(0.5%)	
Unknown	37	(16%)	4	(22%)	9	(16%)	24	(15.5%)	
CA19–9 [kU/I] (mean and SD)	824	1,976	764	1,906	911	2,256	783	1,881	0.720
Pancreatic cancer size									
≤3 cm	114	(50%)	5	(28%)	27	(47%)	82	(54%)	0.341
>3 to ≤5 cm	97	(43%)	12	(66%)	26	(46%)	59	(39%)	
>5 cm	14	(6%)	1	(6%)	4	(7%)	9	(6%)	
Unknown	2	(1%)			0	(0%)	2	(1%)	
Venous invasion									
PV			1	(6%)					NA
SMV			8	(44%)					
PV + SMV			9	(50%)					
Resection									
R0	125	(55%)	7	(39%)	29	(51%)	89	(58.5%)	0.450
R1	94	(41%)	10	(55.5%)	27	(47%)	57	(37.5%)	
R2	6	(3%)	1	(5.5%)	1	(2%)	4	(3%)	
Unknown	2	(1%)	0	(0%)	0	(0%)	2	(1%)	
Cancer stage									
Ia	1	(0.5%)	0	(0%)	0	(0%)	1	(0.5%)	0.167
Ib	10	(4%)	0	(0%)	0	(0%)	10	(6.5%)	
IIa	33	(14.5%)	3	(16.5%)	5	(9%)	25	(16%)	
IIb	167	(74%)	14	(78%)	50	(88%)	103	(68%)	
III	5	(2%)	1	(5.5%)	0	(0%)	4	(3%)	
IV	9	(4%)	0	(0%)	2	(3%)	7	(5%)	
Unknown	2	(1%)	0	(0%)	0	(0%)	2	(1%)	
Chemotherapy									
Neoadjuvant chemotherapy	13	(6%)	5	(28%)	1	(2%)	7	(5%)	0.002
Adjuvant chemotherapy	179	(79%)	13	(72%)	47	(82%)	119	(78%)	0.590
Vital status									
Dead	155	(68%)	14	(78%)	45	(79%)	96	(63%)	0.063
Alive or censored	72	(32%)	4	(22%)	12	(21%)	56	(37%)	
Overall survival [month]									
Median	23.9		22		22.7		24.1		0.256

If not specified, numbers are patients with respective percentages.

PD, pancreatoduodenectomy; PMVR, porto-mesenteric venous resection; SD, standard deviation; CA19-9, carbohydrate antigen 19-9; 95% CI: 95% confidence interval; PV, portal vein; SMV, superior mesenteric vein; OS, overall survival.

### 3.2 Surgical and pathological data

Data are summarized in **Supplementary Material Table 1**.

### 3.3 PMVR with graft interposition subgroup

**Figure 3:**
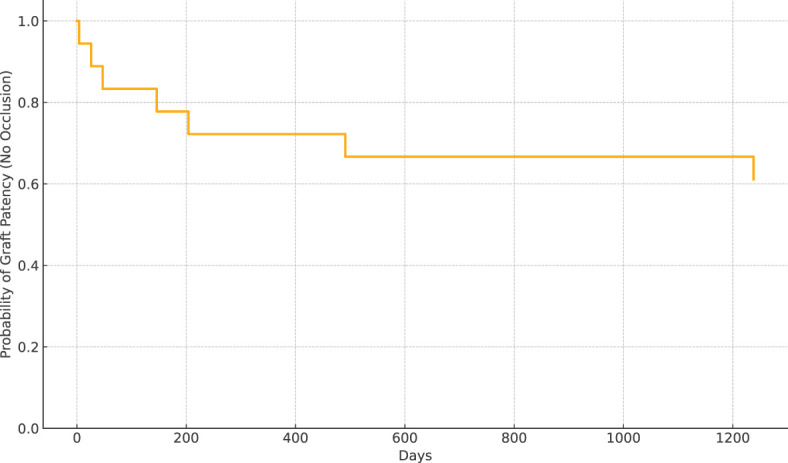
Kaplan–Meier analysis of graft patency over time.

**Figure 4:**
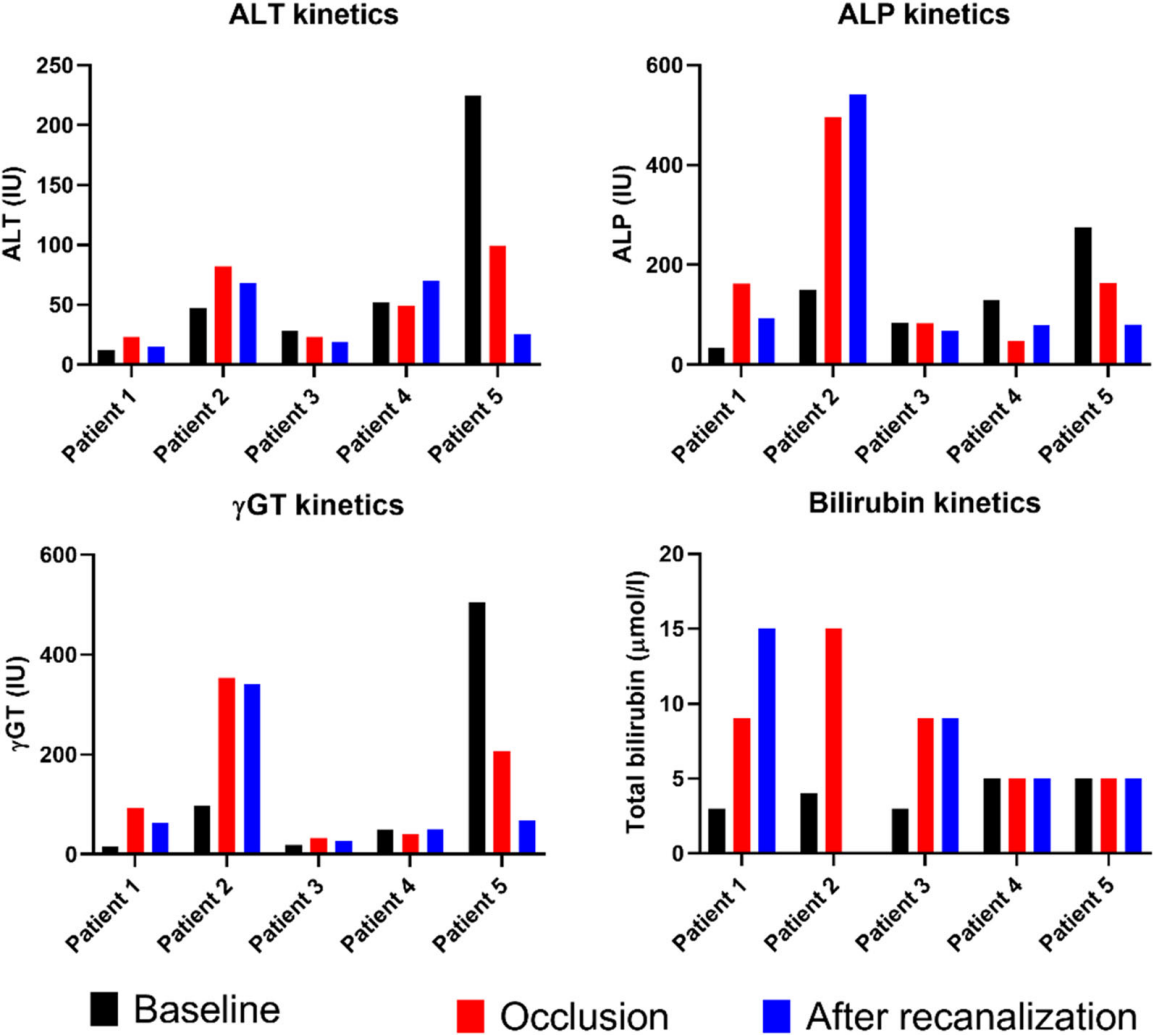
Graphical representation of liver enzyme kinetics at baseline, at occlusion of prosthetic graft, and after recanalization and stenting (measurements performed between 7 and 21 days post-procedure). ALT, alanine aminotransferase; ALP, alkaline phosphatase; γGT, gamma-glutamyltranspeptidase.

**Figure 5:**
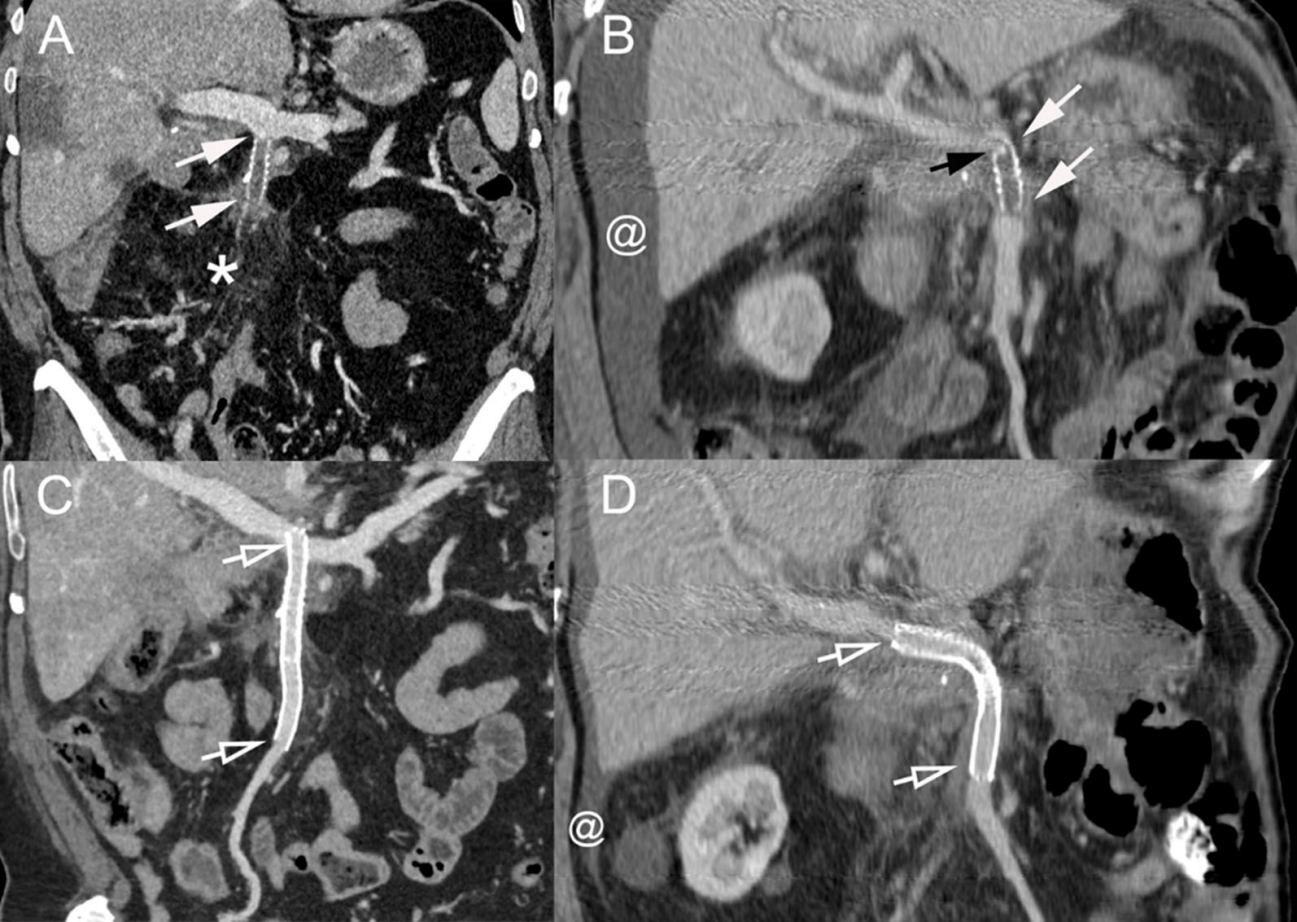
**(A)** Coronal CT image (portal venous phase) of a 60-year-old man with pancreatic cancer treated with PD with distal SMV resection and prosthetic graft placement presenting with asthenia, weight loss, and abdominal pain. The prosthetic graft contains hypoattenuating material consistent with thrombosis (arrows). Fat stranding is also present around the mesenteric vessels and graft (*). **(B)** Coronal CT scan image (portal venous phase) in a 75-year-old man with pancreatic cancer treated with PD with distal SMV resection and prosthetic graft placement presenting with asthenia and abdominal distention due to ascites. The graft is thrombosed (arrows) due to a stenosis at the cranial anastomosis (black arrow). Ascites is present (@). **(C, D)** Coronal CT scan image (portal venous phase) after percutaneous transhepatic recanalization and stenting of the occluded grafts of patients [**(A, B)**, respectively]. Stents are patent (hollow arrows) with disappearance of the fat stranding in panel **(A)** and marked decrease of the ascites in panel **(B)** (@). PD, pancreatoduodenectomy; SMV, superior mesenteric vein.

**Figure 6:**
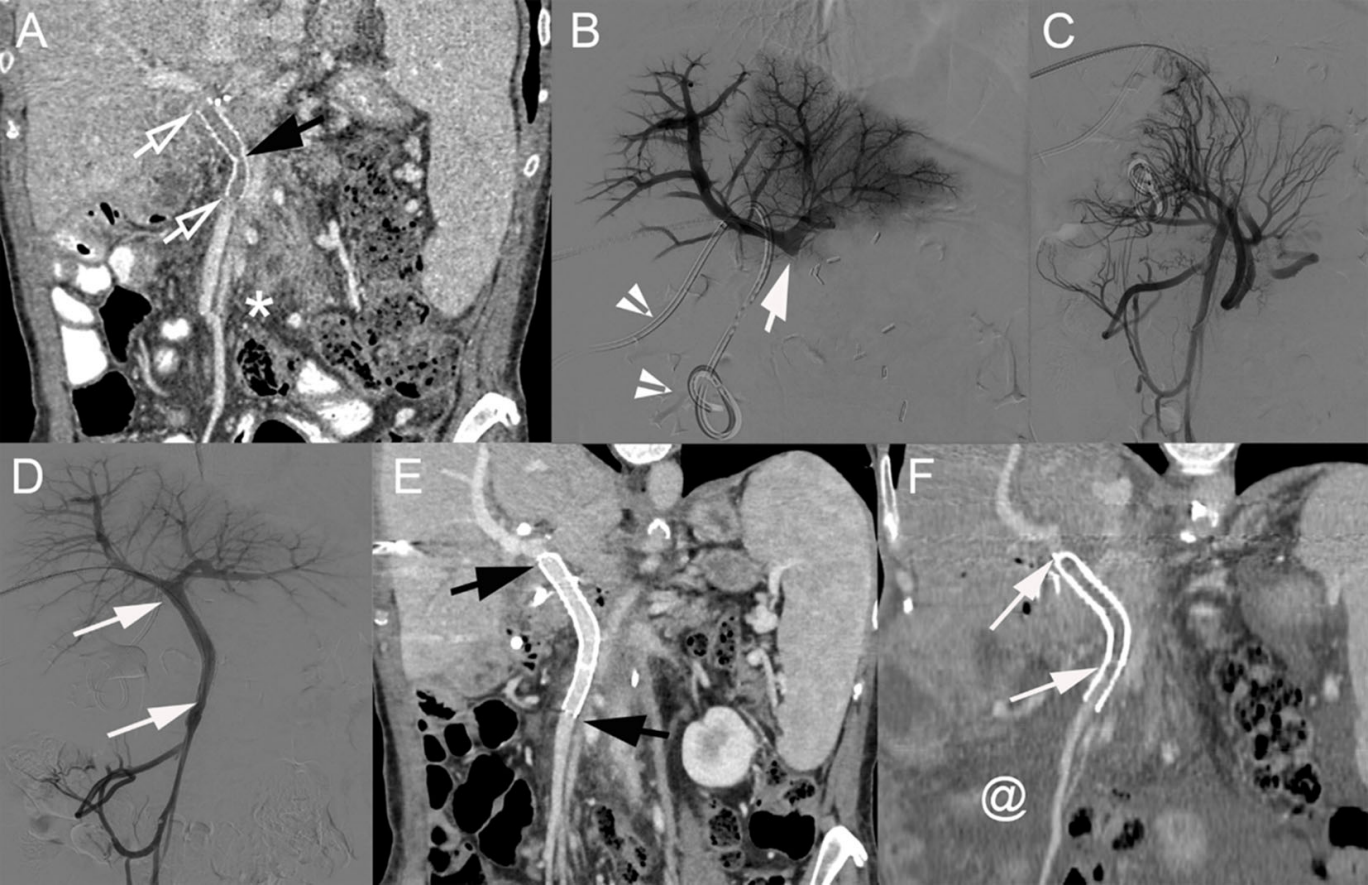
**(A)** Coronal CT image (portal venous phase) of a 60-year-old woman with pancreatic cancer 6 months after PD with venous resection and prosthetic graft placement between the SMV and PV (below the PV bifurcation). The prosthetic graft contains hypoattenuating material consistent with thrombosis (hollow arrows). A kink in the graft is present (black arrow). **(B)** Direct portography showing complete graft occlusion below the PV bifurcation (arrow). Biliary drainage catheter (arrowheads). **(C)** Venography of the caudal SMV (after crossing the thrombosed graft) showing the development of multiple venous collaterals. **(D)** After deployment of 2 stents [8 × 40 mm (SMART Flex, Cordis) and 10 × 60 mm (SMART Control, Cordis); arrows], completion mesenteric portography demonstrates complete recanalization and disappearance of venous collaterals. Coronal CT image (portal venous phase) at 1 week **(E)** and 17 months **(F)** after percutaneous transhepatic venous graft recanalization and stenting. **(E)** Stent patency is visible (black arrows). **(F)** Complete stent thrombosis is demonstrated (arrows) together with the development of marked ascites (@). PD, pancreatoduodenectomy; SMV, superior mesenteric vein; PV, portal vein.

Prosthetic interposition was performed between two segments of the superior mesenteric vein (SMV) (eight patients), the SMV (or one of its main branches), and the PV (nine patients), or between two segments of the PV (one patient). The median length of the prosthetic graft was 3.8 cm (range: 2.8–9). The median diameter was 8 mm (range: 6–16). Seven of 18 patients (39%) had graft occlusion. The median time from PD to graft occlusion was 146 days (range: 4–1,238). Occluded grafts had a diameter of 8–11 mm, while no occlusion occurred in grafts > 11 mm. Patients who experienced graft occlusion had lower maximal diameter on CT than those who did not (8.8 ± 1.1 versus 11.3 ± 1.6 mm, *p* = 0.0034). The size cut-off point was 9.5 mm (sensitivity: 100 [95% CI: 72–100], specificity: 86 [95% CI: 42–100], area under the curve (AUC) 0.93 [95% CI: 0.79–1.00]). Risk factors for surgical graft occlusion are presented in **Table 2**. On univariable logistic regression analysis, small postoperative graft diameter (OR: 0.141; 95% CI 0.021–0.970) and caudal anastomosis diameter measured on CT (OR: 0.226; 95% CI 0.059–0.859) were clear predictors of graft occlusion (*p* = 0.047 and *p* = 0.029, respectively), while cranial anastomosis diameter almost reached significance (*p* = 0.062) (OR: 0.179; 95% CI 0.029–1.092; **Table 2**). On stepwise multivariable logistic regression analysis, only the postoperative caudal anastomosis diameter measured on CT independently correlated with the risk of prosthetic graft occlusion (OR: 0.226, *p* = 0.029; **Table 2**). Occlusion was associated with tumor recurrence in three out of seven patients (43%). The length of the thrombosed graft was similar to that of the patent graft (mean: 4.3 vs. 4.1 cm, respectively; *p* = 0.70). The Kaplan–Meier curve of graft patency is shown in **Figure 3**. Venous graft occlusion was symptomatic in most patients (6/7 patients, 86%) and associated with ascites (4/7 patients), abdominal pain (3/7 patients), cholangitis (1/7 patients), dyspnea (1/7 patients), and hemorrhage from rupture of esophageal varices (1/7 patients). One patient was asymptomatic (1/7 patients, 14%). No consistent change in transaminase levels was observed upon graft occlusion and after recanalization (**Figure 4**). Graft occlusion was detected in all patients on CT (**Figures 5**, **6**).

**Table 2:** Risk factors for prosthetic graft occlusion.

	Univariable logistic regression analysis			Stepwise multivariable logistic analysis		
	Odds ratio	95% CI	*p*-Value	Odds ratio	95% CI	*p*-Value
Neoadjuvant chemotherapy	3.0	0.348–25.8	0.318			
Baseline CA19-9	1.0	0.99–1.00	0.453			
Cancer size	0.337	0.087–1.3	0.114	—	—	—
Tumoral vein stenosis	0.3	0.025–3.626	0.344			
Cranial vein diameter (i.e., to the tumor infiltration)	1.419	0.754–2.672	0.278			
Caudal vein diameter (i.e., to the tumor infiltration)	1.647	0.832–3.262	0.152			
PTFE graft diameter	0.557	0.188–1.650	0.291			
PTFE graft length	1.01	0.948–1.071	0.800			
Portal vein clamping time	0.972	0.922–1.025	0.293			
Postoperative anticoagulant treatment	0.429	0.062–2.972	0.391			
Adjuvant chemotherapy	0.938	0.114–7.728	0.952			
Postoperative maximal graft diameter on CT	0.141	0.021–0.970	0.047	—	—	—
Postoperative cranial anastomosis diameter on CT	0.179	0.029–1.092	0.062	—	—	—
Postoperative caudal anastomosis diameter on CT	0.226	0.059–0.859	0.029	0.226	0.059–0.859	0.029
Splenic vein configuration on CT	1.335	0.225–7.941	0.750			

CA19-9, carbohydrate antigen 19-9; PTFE, polytetrafluoroethylene.

### 3.4 Percutaneous revascularization of thrombosed surgical graft

Of the seven patients with graft occlusion, percutaneous transhepatic endovascular recanalization was attempted in five (72%). Of the two patients in whom no attempt at recanalization was performed, one patient was asymptomatic and the other had a rapidly progressing disease. Data regarding patients who underwent revascularization are summarized in **Table 3**. The median time from graft occlusion diagnosis to recanalization was 9 days (range: 1–41). Recanalization and stenting procedures were feasible and successful in all patients. The median total length and number of stents placed were 9 cm and 2, respectively, with a diameter between 8 and 12 mm (**Table 3**). Immediately before stent placement, IV heparin (60–70 units/kg) was given (four patients). The pressure gradient (above and below the occlusion) was from a mean of 11 ± 1.5 mmHg pre-stenting to 1 ± 1 mmHg post-stenting.

**Table 3:** Data of patients with thrombosed surgical graft and percutaneous revascularization.

PD surgery to occlusion (days)	Occlusion to recanalization (days)	Graft length (mm)	Number of stents placed	Stent location	Total stent length (cm)	Immediate porto-mesenteric flow restoration	2nd occlusion (stent thrombosis)	Time to re-occlusion (days)	Evolution of symptoms after recanalization
47	3	28	4	Jejunal vein–right PV	21	Yes	No	NA	Disappearance of abdominal pain and ascites
491	NA	38	-	-	-	-	-	-	-
146	28	90	2	SMV–PV	9	Yes	Yes	295	Disappearance of ascites
204	41	38	2	SMV–PV	13	Yes	No	NA	Improvement of abdominal pain and resolution of ascites
1,238	1	46	2	Splenic vein–PV	6	Yes	No	NA	No recurrent bleeding (due to portal hypertension related to graft occlusion)
26	9	33	1	SMV–PV	8	Yes	Yes	119	Disappearance of abdominal pain, ascites, and dyspnea
4	NA	25	-	-	-	-	-	-	-

PD, pancreatoduodenectomy; PV, portal vein; SMV, superior mesenteric vein; NA, not applicable.

After recanalization, four patients received anticoagulation (one received low-molecular-weight-heparin for 6 months followed by long-term aspirin, two received acenocoumarol, and one received acenocoumarol/aspirin), while one patient received double anti-platelet therapy for 6 months (clopidogrel/aspirin). The procedure was well tolerated and safe without any complications. One patient developed unrelated ascending cholangitis during the 30-day period following the recanalization. Out of these five patients, two had re-occlusion of their venous prosthesis (time to re-occlusion 119 and 295 days) in the setting of disease progression. One patient had a second interventional revascularization procedure that was successful, and stents remained patent. A second recanalization procedure was not attempted in the second patient because of the rapidly progressing disease. Overall, 4/5 patients (80%) had their stented venous graft open at the end of the follow-up period. The median follow-up after recanalization was 270 days (range: 2–1,545).

Following recanalization and stenting of the occluded graft, symptoms markedly improved in all patients within 7 days, with the disappearance of ascites (four of four patients), dyspnea (one patient), and abdominal pain (two patients). Abdominal pain significantly decreased following recanalization, but low-grade pain persisted in another patient. The patient with variceal rupture stopped bleeding after recanalization, with no bleeding recurrence.

### 3.5 Survival

Survival data of the whole cohort and subgroups are reported in the **Supplementary Material**.

## 4 Discussion

The complexity of pancreatic cancer surgery has increased to expand indications for curative intent resection. PD associated with venous resection and repair by performing direct end-to-end anastomosis or graft interposition has been increasingly performed. This leads to new challenges in the post-surgical follow-up such as the occurrence of prosthetic graft thrombosis.

Prosthetic graft thrombosis can develop both “early” and “late” (with a cut-off defined as 90 days) (7). Similarly, we also observed in our study a timeframe to occlusion ranging from days to years (median: 146 days, range: 4–1,238). This variability in occlusion time underscores the variety of factors that may contribute to thrombosis. It has been suggested that early postprocedural thrombosis is associated with technical issues such as prosthesis kinking, while late thrombosis may more frequently be linked to local tumor recurrence, which was also observed in our study (7). Indeed, all patients who had graft thrombosis after 90 days had concomitant tumor recurrence. However, no predictors of venous thrombosis after PD with PMVR could be identified (7). Neoadjuvant therapy, prolonged operative time, and the use of prosthetic graft were associated with an increased risk of thrombosis after PD with venous resection as reported in another work (8). The use of “Y”-shaped Dacron grafts in case of extended venous resection has also been reported as a potential risk factor for thrombosis (19). In our study, graft and anastomosis diameters were predictors of graft thrombosis, especially small caudal anastomosis diameter. The other factors analyzed (e.g., tumor size, graft length, and neoadjuvant or adjuvant therapy) were not predictors of graft thrombosis. This highlights the importance of selecting the appropriate type of graft depending on the complexity and extent of the venous resection. Among available vessel substitutes, synthetic grafts, peritoneal grafts, or autologous veins are the most frequently used grafts (20–22). Of note, in our center, exclusively prosthetic interposition grafts were used corresponding to our local practice and expertise, while others preferred autologous grafts (23). Although synthetic grafts are considered to have a higher risk of thrombosis, they offer the advantages of being readily available, customizable in terms of size and length, and preventing donor-site morbidity (24).

Regardless of the cause, prosthetic graft thrombosis is symptomatic in most patients with the occurrence in particular of abdominal pain, ascites, or bleeding from variceal rupture due to portal hypertension. Thus, gaining knowledge about the management of these graft occlusions is important. Here, we described an interventional approach to treat graft thrombosis and restore porto-mesenteric blood flow. Technical success was 100% with no immediate/delayed complications. Importantly, after blood flow restoration, patients experienced rapid symptom relief. Of note, if a second graft thrombosis occurs, recanalization and stenting can be safely repeated if needed. The timing of surgery and revascularization should be considered, as surgical anastomoses are fragile after surgery. If PD is recent, we recommend careful small-diameter balloon pre-dilatation of the occluded segment at the level of the anastomoses. The graft diameter and location must be considered. Occluded graft recanalization may be trickier than the recanalization of an occluded native vessel from tumor recurrence. Indeed, the native SMV–PV stenosed/occluded by the tumor may better accommodate a larger stent (10–14 mm depending on location), whereas the stent diameter must not be too large in PD patients with occluded graft, as it may reduce the non-expandable graft lumen. After stenting, if the flow and pressure measurements are adequate, we recommend avoiding vigorous post-stenting angioplasty to prevent graft anastomosis rupture. Of note, in the setting of a graft occlusion, thrombolysis has no role to play in our opinion due to the bleeding risk and the absence of treatment of the underlying cause of thrombosis (e.g., graft kink). Thrombectomy is not needed, as the volume of blood clot is low and limited to the occluded graft.

We observed significant heterogeneity regarding postoperative and long-term anticoagulation/anti-platelet therapy policy after surgery with graft interposition. This is explained by the absence of dedicated guidelines and highlights that the decision to administer anticoagulation/anti-platelet therapy must consider the bleeding risk following surgery and underlying co-morbidities (9, 25). Overall, graft thrombosis occurred in patients with or without anticoagulation or anti-platelet therapy. A systematic review addressing this issue also observed significant heterogeneity in postoperative anticoagulation/anti-platelet therapy after PD with PMVR, which was prescribed in ~50% of patients (9). Since early thrombosis does not seem to be associated with tumor recurrence, it is tempting to speculate that aggressive anticoagulation therapy may prevent graft thrombosis. However, thrombosis occurrence was similar with or without anticoagulation therapy after PMVR (9). Overall, there seems to be insufficient data on whether to support the routine use of anticoagulation/anti-platelet therapy following PD with PMVR, and further research is needed. In our study as well, the anticoagulation regimen was not standardized, which poses a significant challenge in accurately assessing the effect of anticoagulant therapy on vascular patency. Standardizing the anticoagulation protocols would likely improve the consistency of outcomes and allow for more reliable evaluations.

Whether more aggressive surgical management with PMVR improves patient outcomes or increases surgical morbidity and mortality remains widely debated and controversial among different studies and meta-analyses (3, 6, 7, 26). In our study, we found that PMVR was not associated with increased surgical morbidity or 30-day mortality. In addition, surgical margins were not significantly different compared to PD without venous reconstruction. Overall survival was also similar between the groups.

Our study has limitations. Our cohort is relatively small. Indeed, PMVR with prosthetic graft is not frequently performed. However, graft thrombosis is frequent (39% in our series) and can cause significant symptoms. While the present study identified some factors associated with graft occlusion, the limited size of the patient cohort may restrict the generalizability of these findings. Thus, more data about safety and efficacy are needed, as effective management is fundamental for these fragile patients. Our results need to be confirmed in a larger prospective series.

In conclusion, percutaneous transhepatic prosthetic graft recanalization and stenting is feasible and may be considered safe and effective with immediate restoration of porto-mesenteric blood flow and symptom relief. Small grafts and anastomosis diameters are particularly at risk of thrombosis.

## Data Availability

The raw data supporting the conclusions of this article will be made available by the authors, without undue reservation.
