# Supplementary material for: Percutaneous transhepatic recanalization of occluded prosthetic graft after pancreatoduodenectomy with venous reconstruction for pancreatic cancer
Source: Front Oncol. 2025 Jul 17;15:1575481. doi: 10.3389/fonc.2025.1575481 (PMC12310483; doi:10.3389/fonc.2025.1575481)
Supplement: Supplementary file 1 [file DataSheet1.docx]

1. Supplemental data

Surgical technique

Patients were discussed at a multidisciplinary tumor board and gave their written informed consent the surgery. Patients were operated by experienced surgeons specialized in pancreatic surgery and done according to standard procedures. In addition, venous resection was performed if venous tumor infiltration was observed on preoperative imaging or suspected intraoperatively. The technique of venous reconstruction depended on the length of venous involvement by the tumor. For short resected vein, direct end-to-end anastomosis was the preferred technique. If the vein to be resected was too long for primary anastomosis a prosthetic graft (polytetrafluoroethylene) was used. No autologous grafts were used. After surgery, prophylactic intravenous heparin was administered, followed by prophylactic subcutaneous low-molecular-weight heparin until discharge. Graft patency was assessed on imaging during the postoperative stay by CT-scan.

Results

Surgical data

Procedure was the longest in patients who required PVMR and graft interposition (437+/-83 minutes), while it was the shortest in patients who underwent PD without venous resection (333+/-60 minutes), (*p*=0.0001). Mean blood loss was 543+/-461 mL in patients who did not require vein resection while it was 758+/-519 mL in patients with PVMR and end-to-end anastomosis. Graft interposition was associated with the highest blood loss (1409+/-679 mL) (**Supplemental Table 1**) compared to PVMR with end-to-end-anastomosis (*p*<0.001) and PD without venous resection (*p*<0.001).

Postoperative Morbidity, graded according to the Clavien-Dindo classification, hospital stay and 30-day mortality was comparable among subgroups (all: *p*≥0.61; **Supplemental** **Table 1**). Importantly, the patient who died in the prosthetic graft subgroup underwent an exploratory laparoscopy 48 hours after initial surgery because of unfavorable clinical evolution. Prosthetic graft was not occluded but was revised during this second intervention because of diminished hepatic portal venous flow secondary to partial kinking of the graft. The patient eventually died 72 hours later because of sceptic chock and multiorgan failure.

*Pathological data*

Among patients who underwent PD with PMVR and prosthetic graft interposition, 1 (5.5%),16 (89%) and 1 (5.5%) patient(s) had a T2, T3 and T4 tumor, respectively. 15 patients (83%) had positive lymph nodes and resection was R0 in 39%, R1 in 55.5%, R2 in 5.5%. Cancer stage was classified as IIb in 14 patients (78%) (**Table 1**) and was similar among subgroups (all *p*=0.167).

Patients who underwent PD without venous resection demonstrated positive lymph nodes in 75% of the cases, which was significantly lower than for patients with PD and PVMR with end-to-end anastomosis (91%) (*p*=0.019). No difference in nodal status was observed in patients who underwent PD without PMVR vs. PMVR and prosthetic graft interposition (N1 75% vs. 83%; *p*=0.66). No difference was observed between PD and PMVR with prosthetic graft versus end-to-end anastomosis (N1 83% vs. 91%, *p*=0.39) (**Supplemental Table 1**). There was no difference in surgical margins (R0-R1-R2) between groups with or without venous resection (*p*>0.26) or among subgroups (*p*=0.45, **Table 1**).

*Survival*

The median OS of the whole cohort was 23.9 months. Survival was similar between PD alone vs PMVR with end-to-end anastomosis and prosthetic graft interposition (24.1 vs 22.7 months, respectively [HR 0.76, 95%CI: 0.54-1.1; *p*=0.102]) or between the PMVR end-to-end anastomosis vs prosthetic graft (22.7 vs 22 months, [HR 0.93, 95%CI: 0.5-1.7; *p*=0.8]). No difference in survival time was observed between the 3 groups (*p*=0.256) (Supplemental Figure 1).


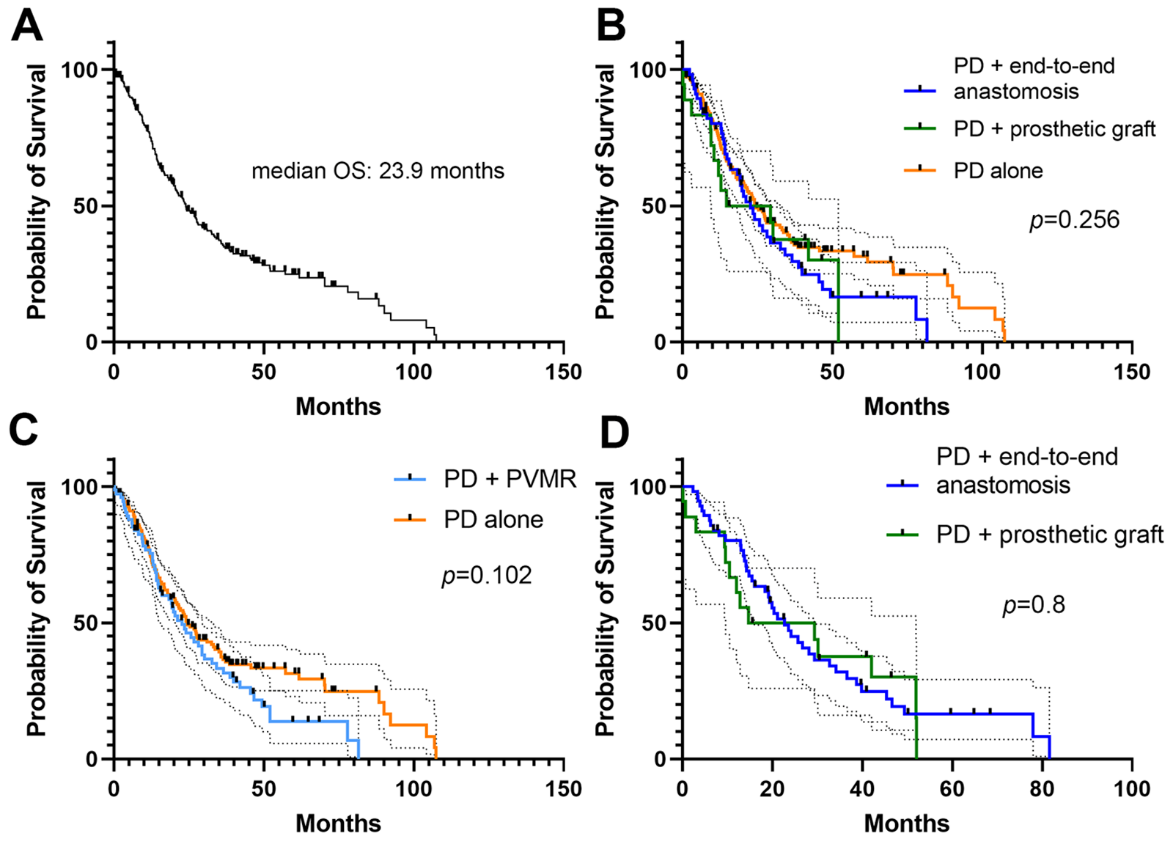


**Supplemental Figure 1.** Survival analysis of the whole cohort (A), according to the 3 groups (B), to the PD alone vs PMVR (i.e. end-to-end anastomosis and prosthetic graft) groups (C) and to the PMVR with end-to-end anastomosis vs PMVR prosthetic graft groups.

| **Supplemental Table 1. Pancreatic Cancer Patients Characteristics: surgical and pathological data** | | | | | | | | |  | |
| --- | --- | --- | --- | --- | --- | --- | --- | --- | --- | --- |
|  | **Full cohort** | | **PD + PMVR with**  **prosthesis graft (N=18)** | | **PD + PMVR with**  **end-to-end anastomosis (N=57)** | | **PD without PMVR**  **(N=152)** | | ***p*-value** | |
|  | **(N=227)** | |  |  |  |  |  |  |  |  |
| **Peri-operative data** |  |  |  |  |  |  |  |  |  |  |
| Blood loss [mL] (mean & SD) | 671 | 550 | 1409 | 679 | 758 | 519 | 534 | 461 | <0.001 |  |
| PD time [min] (mean & SD) | 354 | 78 | 437 | 83 | 385.5 | 90.4 | 333 | 60 | <0.001 |  |
| **Post-operative hospital stay** [days] (mean & SD) | 22 | 17 | 18 | 9 | 21 | 15 | 23 | 18 | 0.620 |  |
| **Perioperative mortality** (within 30 days of PD) | 6 | (2.6) | 1 | (5.6) | 1 | (1.7) | 4 | (0.7) | NA |  |
| Tumor stage |  |  |  |  |  |  |  |  | 0.560 |  |
| T1 | 3 | (1%) | 0 | (0%) | 0 | (0%) | 3 | (2%) |  |  |
| T2 | 25 | (11%) | 1 | (5.5%) | 5 | (9%) | 19 | (12%) |  |  |
| T3 | 192 | (85%) | 16 | (89%) | 52 | (91%) | 124 | (82%) |  |  |
| T4 | 5 | (2%) | 1 | (5.5%) | 0 | (0%) | 4 | (3%) |  |  |
| Unknow | 2 | (1%) | 0 | (0%) | 0 | (0%) | 2 | (1%) |  |  |
| **Positive lymph nodes** |  |  |  |  |  |  |  |  | 0.082 |  |
| Yes | 181 | (80%) | 15 | (83%) | 52 | (91%) | 114 | (75%) |  |  |
| No | 44 | (19%) | 3 | (17%) | 5 | (9%) | 38 | (25%) |  |  |
| Unknown | 2 | (1%) | 0 | (0%) | 0 | (0%) | 0 | (0%) |  |  |
| **Postoperative morbidity (Clavien-Dindo)** |  |  |  |  |  |  |  |  | 0.770 |  |
| 0 | 59 | (26%) | 4 | (22%) | 14 | (24.5%) | 41 | (27%) |  |  |
| 1 | 10 | (4%) | 2 | (11%) | 2 | (3%) | 6 | (4%) |  |  |
| 2 | 69 | (31%) | 7 | (39%) | 18 | (31.5%) | 44 | (29%) |  |  |
| 3a | 47 | (21%) | 3 | (17%) | 14 | (24.5%) | 30 | (20%) |  |  |
| 3b | 16 | (7%) | 0 | (0%) | 6 | (10.5%) | 10 | (6.5%) |  |  |
| 4a | 10 | (4%) | 1 | (5.5%) | 1 | (2%) | 8 | (5%) |  |  |
| 4b | 10 | (4%) | 0 | (0%) | 1 | (2%) | 9 | (6%) |  |  |
| 5 | 6 | (3%) | 1 | (5.5%) | 1 | (2%) | 4 | (2.5%) |  |  |
| Abbreviations: PD, pancreatoduodenectomy; PMVR, porto-mesenteric venous resection; SD, standard deviation; CA19-9, Carbohydrate antigen 19-9 ; 95%CI: 95% confidence interval ; PV, portal vein; SMV, superior mesenteric vein; OS, overall survival | | | | | | | | | | |
| Note: if not specified numbers are patients with respective percentages | | | | | | | | | | |
